# An Immunohistochemical Study on the Expression of Sex Steroid Receptors in Canine Mammary Tumors

**DOI:** 10.5402/2012/378607

**Published:** 2012-02-15

**Authors:** Leena Rajathy Port Louis, Khub Chandra Varshney, Madhavan Gopalakrishnan Nair

**Affiliations:** Department of Veterinary Pathology, Rajiv Gandhi College of Veterinary and Animal Sciences, Kurumbapet, Puducherry 605 009, India

## Abstract

Steroid hormones are found to play a major role in the genesis and progression of mammary tumors. The aim of this study was to immunohistochemically detect the presence of estrogen receptor alpha (ER**α**), estrogen receptor beta (ER**β**), and progesterone receptor (PR) and also to study the association between these markers in 29 cases of benign (11) and malignant (18) canine mammary tumors. ER**α** immunostaining was noticed in only one case of carcinosarcoma specifically in the nuclei of epithelial and a few myoepithelial cells. ER**β** immunostaining was noticed in the nuclei and cytoplasm of epithelial cells and smooth muscles lining the blood vessels. Immunoexpression of ER**β** was 82% in benign tumors and 78% in malignant tumors. PR immunostaining was expressed in the nuclei of epithelial cells in both benign and malignant tumors. Among the 15 PR+ cases, 6 (55%) were of benign type, and 9 (50%) were of malignant type. The most common group of hormone receptor was the ER**α**−/PR+/ER**β**+ (46%) in benign tumors and ER**α**−/PR−/ER**β**+ (38%) in malignant tumors. Although there was no significant association between ER**α** and PR with ER**β**, the findings indicated that ER**β** was consistently expressed in both benign and malignant tumors, irrespective of ER**α** and PR status.

## 1. Introduction

Mammary gland tumors are among the most common neoplasms in dogs and have been reported worldwide. The majority of mammary neoplasms in female dogs are of epithelial origin, and approximately 50% of them are malignant [1]. These neoplasms have a complex morphology in forming epithelial, mixed, and mesenchymal types [2]. Histologic evidence of cancer does not invariably imply a malignant clinical course. Therefore, reliable prognostic factors are of great importance for estimating the individual risk of unfavourable clinical outcome. There are some recognized, well-accepted prognostic factors of malignant mammary neoplasms in the dog, and these include tumor size, lymph node status, histologic type, histologic malignancy grade, degree of nuclear differentiation, and distant metastasis. There are also other proven or controversial host and tumor prognostic factors such as HER-2, p53, PCNA, and Ki-67, and the number of new ones are steadily increasing [3]. Although several studies have been carried out on the prognostic aspects of canine mammary neoplasms, some areas, especially the role of steroid hormone receptors, remain uncertain [4]. The risk of canine mammary neoplasia is affected by exposure to estrogen in early mammary development. The relative risk for mammary neoplasms in female dogs spayed before estrous is 0.5%, after the first cycle 8%, 26% after the second cycle; with the protective effect being lost after about 4 cycles. Inspite of this, there are only limited reports on clinical utilization of proliferation markers and steroid receptors in canine mammary neoplastic conditions [5]. Both estrogen and progesterone are mitogenic by autocrine or paracrine mechanisms. Since steroids regulate the expression of certain cyclins or kinase inhibitors, they may control cell-cycle progression directly [6].

The development of most mammary gland carcinomas is estrogen dependent, and the majority of canine mammary gland carcinomas express estrogen receptors (ERs). Benign tumors and well-differentiated tumors are more likely to be ER-positive, whereas undifferentiated, anaplastic tumors are more likely to be ER-negative [4]. Estrogen receptor expression has also been found to be associated with the hormonal status of the dog. Younger, intact dogs were more likely to have ER-positive tumors than older ovario-hysterectomized dogs.

In contrast to the normal breast, most premalignant breast lesions express high levels of ER*α*, and ER*α*-expressing breast cancer cells are hormone dependent and undergo regression when estrogen is removed [7]. Thus ER*α* is a well-established predictive marker of hormone sensitivity in breast cancer as well as a positive prognostic marker. In addition to being involved in the initial malignant transformation, the ER may also represent a rationale therapeutic target in canine mammary gland neoplasms, as in breast cancer of women [8]. In a study on immunohistochemical detection of ER*α* in a canine mammary tumor [4], it was reported that ER*α* immunostaining was localized in the nuclei of normal, benign, and malignant epithelial and also in myoepithelial cells, whereas the normal and neoplastic stromal cells, cartilaginous cells, and bone cells in mixed neoplasms were negative.

Martín De las Mulas and coworkers [9] studied the immunohistochemical detection of ER*β* in normal and tumor-affected canine mammary glands. They reported the expression of ER*β* in the nuclei of acinar and ductal epithelial cells and some basal myoepithelial cells. Myoepithelial cells of the complex and mixed tumors, chondrocytes of mixed tumors, and sarcoma cells were negative. Benign mixed and complex neoplasms had a higher expression of the ER*β* than simple neoplasms, suggesting that ER*β* expression may be a favorable prognostic factor because the grade of malignancy of complex carcinomas is lower than that of simple carcinomas. However, the data with regard to ER*β* expression in invasive neoplasms of the human breast and its relationship to prognosis are somewhat contradictory, with some groups reporting that the presence of this receptor is a good prognostic factor and others reporting the reverse. Therefore, the role played by ER*β* in mammary neoplasms is not clear. It was suggested that ER*α* and ER*β* play different roles in cell proliferation and carcinogenesis of breast cancer, partly by mediating the transcription of various genes via different types of deoxyribonucleic acid enhancers. Experimental studies indicated that ER*β* may modulate ER*α* transcriptional activity. Therefore, ER*β* determination may provide additional information on the responsiveness of canine mammary carcinomas to different endocrine treatments. Considering these factors, it was suggested that the ratio between ER*α* and ER*β* expression in canine mammary neoplasms may be useful to identify subgroups of ER-positive neoplasms [9].

Progesterone is essential for the development and growth of the mammary glands, but it also increases the risk of development of mammary neoplasia. Mechanisms involved in progesterone-induced mammary gland neoplasms include an upregulation of growth hormone (GH) production within the mammary gland [8]. Progesterone receptor (PR) status is a marker for hormone responsiveness and disease prognosis in breast cancer. PR-negative neoplasms generally have poor prognosis than progesterone receptor-positive neoplasms [10]. Positive PR staining has been reported in the nucleus of normal and neoplastic epithelial cells as well as in myoepithelial cells of normal and neoplastic canine mammary tissues. In addition, cytoplasm of the spindle as well as stellate cells of complex and mixed tumors was also reported to be immunoreactive to PR [3].

ER*α* and PR receptors are present in more than 50% of mammary neoplasms in dogs [4]. In addition to their ability to predict the response to hormonal therapy, ER and PR status also aids in differentiation of the neoplasm, thereby aiding assessment of patient prognosis [3].

The aim of the present study was to immunohistochemically detect the presence of ER*α*, ER*β*, and PR receptors in benign and malignant canine mammary tumors and also identify the association between these markers.

## 2. Materials and Methods

### 2.1. Tissue Samples

Mammary tumor samples (29 numbers) were obtained by excisional biopsy (mastectomy or nodulectomy) from dogs presented at the teaching hospital of Rajiv Gandhi College of Veterinary and Animal Sciences, (RAGACOVAS), veterinary dispensaries, and other private veterinary practitioners in and around Puducherry, south India. After detailed gross examination, representative tissue samples were fixed in 10% neutral-buffered formalin [11].

### 2.2. Histopathology

The tissue samples fixed in 10% neutral-buffered formalin were processed for histopathological examination by routine paraffin-embedding technique and microtomy [11]. Five-micron thick sections were stained by haematoxylin and eosin method [11], and the tumors were classified according to the WHO histological classification of canine mammary neoplasms [12].

### 2.3. Immunohistochemistry (IHC)

Immunohistochemical detection of ER*α*, ER*β*, and PR was performed on 29 formalin-fixed, paraffin-embedded tissue samples representing both benign and malignant neoplasms. IHC assays were performed on 4-*μ*m sections of formalin-fixed, paraffin-embedded tissue samples. The ready-to-use monoclonal mouse anti-human ER*α* clone 1D5 (Biogenex, USA) and the polyclonal ready-to-use mouse anti-human ER*β* (Biogenex, USA) were used with a Super Sensitive Polymer-HRP Detection System (BioGenex, USA). Dewaxed and rehydrated sections were subjected to heat antigen retrieval by microwaving in 0.01 M citrate buffer, pH 6.0 for 30 minutes at 640 W (80% power). The ready-to-use mouse anti-human PR Clone PR88 (Biogenex, USA) was used with the Super Sensitive Polymer-HRP Detection System (BioGenex, USA). Dewaxed and rehydrated sections were subjected to heat antigen retrieval by microwaving in citrate buffer 0.01 M, pH 6.0 for 10 minutes at 800 W plus two periods of 10 minutes at 320 W. All further IHC-staining procedures were carried out according to the instructions in the test kits. Tissue sections were counterstained with Mayer's hematoxylin. Formalin-fixed, paraffin-embedded tissue sections of canine uterus and human breast cancer were run as positive controls in the standardization of the techniques. The substitution of the specific primary antibodies by Tris-buffered saline (TBS) in tissue sections served as negative control. Classification of staining data was executed semiquantitatively by examining the entire tumor present in the tissue section using immunoperoxidase scoring pattern. The percentage of tumor cells with positive staining (proportional score) was recorded. The intensity of immunohistochemical staining (intensity score) was scored as mild (+), moderate (++), and abundant (+++).

### 2.4. Statistical Analysis

The immunohistochemical data of each parameter was compared using Mann-Whitney U test. Chi-square test (*χ*2) was applied to study the association between ER*α*, ER*β*, and PR expression in benign and malignant tumors. For all the statistical analyses, the level of critical significance was *P* < 0.05 [13].

## 3. Results

### 3.1. Histopathology

Based on histopathological findings, the tumors were categorised as benign (11 cases) and malignant (18 cases). The benign tumors were adenoma (2), duct papilloma (1), fibroadenoma (2), and benign mixed tumor (6). The malignant tumors were papillary adenocarcinoma (2), papillary cystadenocarcinoma (5), solid adenocarcinoma (3), complex adenocarcinoma (3), carcinosarcoma (2), sebaceous adenocarcinoma (1), squamous cell carcinoma (1), and hemangiopericytoma (1).

### 3.2. Immunohistochemistry

In the positive controls, localization of ER*α*, ER*β*, and PR was restricted to the nucleus of cells in all cases of positivity (surface and glandular epithelial cells, stromal fibroblasts of the endometrium, smooth muscle cells of the myometrium, and human breast carcinoma cells). In the negative controls, all nuclei were negative. Immunostaining patterns of ER*α*, ER*β* and PR in various benign and malignant mammary tumors are represented in Table 1.

### 3.3. ER*α* Staining

**Figure 1:**
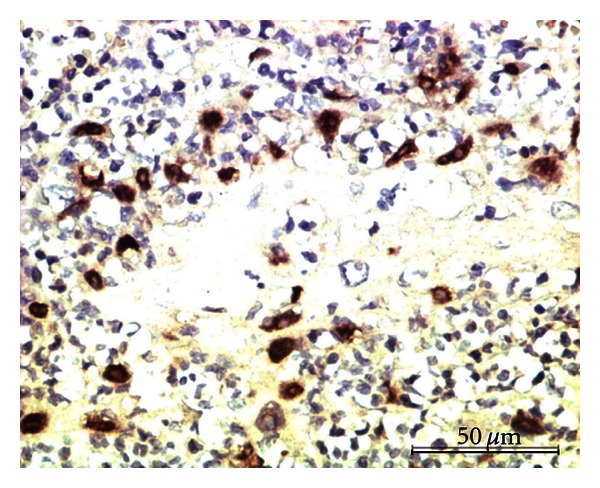
Carcinosarcoma showing the expression of ER*α* in the nuclei of epithelial and myoepithelial cells. Immunoperoxidase/DAB substrate/Mayer's haematoxylin counterstain x400.

ER*α* staining was observed as a brown nuclear staining in the tumor tissue sections. Out of 29 samples analyzed, only one case of carcinosarcoma showed strong positive signals for ER*α*, in the nuclei of both proliferating myoepithelial and epithelial cells (Figure 1). All the other neoplasms (benign and malignant) were negative.

### 3.4. ER*β* Staining

**Figure 2:**
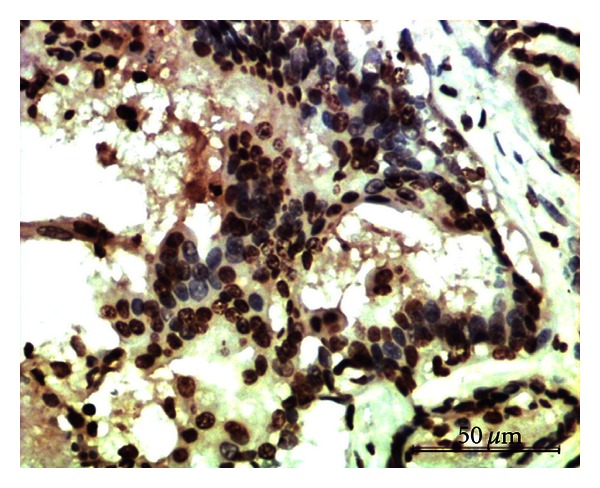
Mixed mammary tumor showing strong expression of ER*β* in the nuclei of epithelial cells. Immunoperoxidase/DAB substrate/Mayer's haematoxylin counterstain x400.

**Figure 3:**
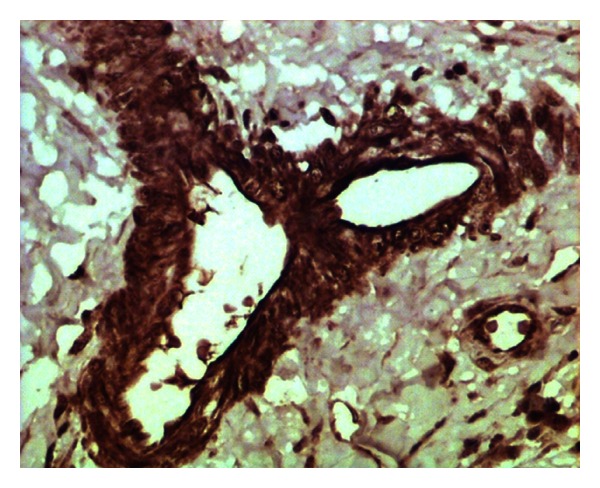
Fibrocystadenoma showing ER*β*-abundant expression in the endothelium and smooth muscles lining the blood vessels. Immunoperoxidase/DAB substrate/Mayer's haematoxylin counterstain x400.

Out of 29 samples analyzed for ER*β* immunostaining, 23 cases (79%) expressed positive signals. Strong nuclear (Figure 2) and weak cytoplasmic staining were observed in glandular and ductular epithelial cells. The vascular endothelium and smooth muscles lining the blood vessels also gave strong signals (Figure 3). ER*β*-staining intensity was abundant in 17%, moderate in 34%, and mild in 28% of the cases.

Immunoexpression of ER*β* in case of benign tumors was 82% (9/11), and in malignant tumors it was 78% (14/18). Among the benign tumors, duct papilloma had the highest ER*β* expression, and among the malignant tumors the maximum expression was noticed in a case of hemangiopericytoma. There was no significant difference (*P* > 0.05) in ER*β* expression between benign and malignant tumors.

### 3.5. PR Staining

**Figure 4:**
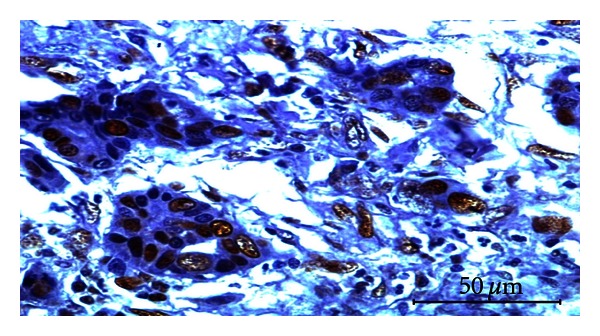
Fibroadenoma showing PR expression in the nuclei of epithelial and stromal cells. Immunoperoxidase/DAB substrate/Mayer's haematoxylin counterstain x400.

Out of 29 samples subjected for PR immunostaining, 15 cases (52%) showed positive signals in the nuclei of neoplastic alveolar and ductal epithelial cells (Figure 4). Very few stromal cells expressed positive immunosignals for PR. Distribution of PR- staining intensity in mammary tumors indicated that 28% of the tumors expressed abundant intensity (+++) followed by 17% of moderate (++) and 7% of mild (+) type.

Out of the 15 PR positive cases, 6 (55%) were of benign type, and 9 (50%) were of malignant type. Among the benign tumors, the highest expression was noticed in benign mixed tumor, and, among the malignant tumors, papillary adenocarcinoma expressed the maximum immunostaining. There was no significant difference (*P* > 0.05) in PR expression between benign and malignant tumors.

### 3.6. Combined Expression of Steroid Receptors

Expression of ER*α* was noticed only in a solitary case of carcinosarcoma, but it was negative for PR. In both benign and malignant tumors, PR and ER*β* expression was noticed. The most frequent combination recorded in benign tumors was ER*β*+ PR+, whereas it was ER*β*+ PR− in malignant tumors. Chi square (*χ*2) analysis revealed no significant association between the distribution of ER*α* and PR with tumor type (benign or malignant). The combined expressions of ER*α*, PR, and ER*β*, PR in canine benign and malignant mammary tumors are represented in Tables 2 and 3, respectively.

### 3.7. Association of ER*β* with ER*α* and PR Status in Benign and Malignant Tumors

The associated expression of ER*β* with ER*α* and PR is represented in Tables 4 and 5, respectively.

The most common receptor expression in benign tumors was ER*α*−/PR+/ER*β*+ combination and in malignant tumors it was ER*α*−/PR−/ER*β*+ combination. Chi square (*χ*2) analysis indicated no significant association between these receptor combinations.

## 4. Discussion

Mammary gland tumors account for more than 50% of all tumors in the female dog [1]. About 65% of mammary tumors in dogs are benign mixed tumors, and 25% are carcinomas. Steroid hormones have been reported to play a key role in the development of mammary neoplasms [8].

The role of ovarian hormones has been well established in human breast cancer studies [14]. In dogs and cats, the involvement of steroid hormones in the development of mammary carcinomas is supported by the protective effects of ovariectomy. Intact females have four times greater risk of getting mammary tumors than the neutered females [15]. Although steroid hormone receptors have been described in canine and feline mammary tumors 25 years ago [1], studies on the role of steroid hormone receptors in the development of mammary tumors in these species are scarce. Progesterone and estrogen receptors are present in both normal and neoplastic tissues. Ninety-five percent of normal canine mammary tissues contain progesterone and/or estrogen receptors. Over 50% of canine mammary tumors express estrogen and progesterone receptors [16].

Progesterone and estrogen have a crucial role in the control of mammary gland proliferation and tumor formation. In dogs, prolonged exposure of the bitch to progesterone stimulates proliferation of the mammary epithelium. The physiological effect of these hormones is mediated mainly by receptors expressed in the mammary tissue [16]. The estrogen receptor (ER) is a regulator of mammary epithelial growth, proliferation, and differentiation whose complex cellular interactions are mediated by a multitude of ligands, cofactors, and other stimuli. ER is important for normal breast development and function but also plays a role in the development and progression of breast cancer. In the clinical setting, breast cancer patients with an ER-positive status have the greatest likelihood of responding to endocrine therapies [17]. In 1996, the existence of a second related ER subtype, ER*β*, was reported [18]. ER*α* and ER*β* genes are present on different chromosomes with the greatest homology (close to 100%) in their DNA-binding domains [19]. The results of some studies have suggested that ER*α* and ER*β* are coexpressed in most breast cancers, but there is evidence that a more complex relationship between these two molecules exists [20]. Recent data have indicated that ER*β* expression is higher in premalignant than in invasive disease and is higher in lobular compared with ductal carcinomas in humans [21]. Further evidence suggests that loss of ER*β* expression correlates with increased tumor aggressiveness in breast cancers [14]. Estrogens are known to bind ER*β* with affinity similar to ER*α*, and the transcriptional activation via the estrogen response element (ERE) is identical for both receptor forms. ER*α* and ER*β* can also form biologically functional receptor heterodimers in tissues in which they are coexpressed.

In the present study, the expression of estrogen (ER*α*, ER*β*) and progesterone receptors were studied in canine mammary tumor tissue sections using immunohistochemistry. The expression of ER*α* was observed only in a solitary case of carcinosarcoma, and all the other 28 neoplasms (benign/malignant) were negative. In earlier studies on canine mammary tumors, a significantly higher expression of ER*α* was recorded in benign tumors compared to malignant tumors [4, 5].

In the literature, there are very few reports available on the activity and expression of ER*β* in canine mammary neoplasms [9, 22]. In the present study, ER*β* expression was observed in both benign and malignant mammary tumors. Immunoreactive products to ER*β* were found markedly in the nuclei of glandular epithelial-type cells. Occasionally, a faint cytoplasmic staining of the glandular epithelial cells was also observed. No immunoreactivity was noticed in the myoepithelial-type cells of complex and mixed tumors, chondrocytes of mixed tumors which was in agreement with an earlier report [9]. Immunoexpression of ER*β* in case of benign tumors was 82% (9/11), and in malignant tumors it was 78% (14/18). ER*β* expression in case of benign tumors was highest in duct papilloma. The maximum immunostaining among the malignant tumors was noticed in a solitary case of hemangiopericytoma. A higher expression of ER*β* in benign tumors compared to malignant tumors has been reported in dogs [9].

In the present study, PR was expressed in the nuclei of epithelial cells in both benign and malignant tumors, whereas the cartilage and bone cells were negative which concorded with an earlier report [23].

The present study also revealed that there was no significant difference in PR expression between benign and malignant tumors. This finding was in contradiction to an earlier report [16] wherein they reported that there was a significant difference in the PR expression between benign and malignant mammary tumors. This discrepancy could be attributed to the less number of mammary tumor cases analyzed in the present study.

Analyses of classical ER and PR have become accepted and useful tools in the prognosis and prediction of hormonal therapy response in human breast cancer [7]. ER*α* and PR receptors are present in more than 50% of mammary neoplasms in dogs [4]. In addition to the ability to predict the response to hormonal therapy, ER and PR status also aids in differentiation of the neoplasm, thereby aiding assessment of patient prognosis [3].

In the present study, the most frequent hormone receptor combination observed was ER*α*−/PR+ combination in 54.55% of benign and 50% of the malignant tumors. However, 44.44% of the malignant tumors were negative for both ER*α* and PR receptor expression. A study reported that the most frequent hormone receptor combination in benign neoplasms was ER*α*+ PR+ group and ER*α*− PR+ among the malignant ones [3].

With respect to ER*β* and PR, the most frequent hormone receptor combination in the present study among benign tumors was ER*β*+/PR+ group (45.45%) and among the malignant neoplasms ER*β*+/PR- group (44.44%). There are no comparative studies in canine mammary neoplasms on the status of ER*β* with PR expression; however, human breast cancer studies have recorded a higher expression of ER*β* with PR in malignant cases [14].

In the present study, the association of ER*α* and PR with ER*β* was assessed. The most common hormone receptor combination observed was ER*α*−/PR+/ER*β*+ combination (46%) in benign tumors and ER*α*−/PR-/ER*β*+ combination (38%) in malignant tumors. Although there was no significant association between ER*α* and PR with ER*β* in both benign and malignant tumors, these findings indicated that ER*β* was consistently expressed irrespective of the ER*α* and PR status. There are no comparative studies in canine mammary tumors on the association of ER*α* and PR with ER*β* status. Coexpression of ER*β* with ER*α* and PR, as well as its association with the other indicators of low biological aggressiveness of breast cancer indicated that ER*β*-positive tumors were likely to respond to hormonal therapy [14].

Canine mammary tumors are challenging for both clinicians and pathologists because the tumors are difficult to fully characterize; thus, their behaviour and prognosis are difficult to predict. However, prognosis and therapy of mammary tumors can be done based on the hormonal receptor expression profiles of the neoplasm as has been practiced in human breast cancer studies. Tumors which are positive for either ER (ER*α*/ER*β*) or PR or both, have a better prognosis than those that are negative for both the receptors. Prognostic power, feasibility, economy, reproducibility, and, if possible, its applicability without highly sophisticated equipments should determine the introduction of new diagnostic methods in clinical oncology.

## Figures and Tables

**Table 1:** Immunostaining percentage (%) and staining intensity (I) of ER*α*, ER*β*, and PR in different canine mammary tumors.

S1.No.	Tumor type	ER*α*% (I)	ER*β*% (I)	PR% (I)
1	Papillary cystadenoma	Negative	10.7 (+)	Negative
2	Fibrocystadenoma	Negative	33.6 (+)	44.4 (++)
3	Duct papilloma	Negative	57.2 (++)	18.5 (+)
4	Fibroadenoma	Negative	47.1 (+++)	Negative
5	Fibroadenoma	Negative	36.1 (++)	51.4 (++)
6	Mixed mammary tumor	Negative	Negative	53.5 (+++)
7	Mixed mammary tumor	Negative	25.2 (+)	Negative
8	Mixed mammary tumor	Negative	36.8 (++)	51.9 (+++)
9	Mixed mammary tumor	Negative	Negative	Negative
10	Mixed mammary tumor	Negative	57.6 (++)	60.2 (+++)
11	Mixed mammary tumor	Negative	79.6 (+++)	Negative
12	Papillary adenocarcinoma	Negative	10.5 (++)	Negative
13	Papillary adenocarcinoma	Negative	Negative	61.9 (+++)
14	Papillary cystadenocarcinoma	Negative	Negative	Negative
15	Papillary cystadenocarcinoma	Negative	15.9 (++)	60.5 (+)
16	Papillary cystadenocarcinoma	Negative	Negative	46.4 (++)
17	Papillary cystadenocarcinoma	Negative	11.3 (++)	Negative
18	Papillary cystadenocarcinoma	Negative	11.1 (+)	72.5 (+++)
19	Solid adenocarcinoma	Negative	48.5 (++)	17 (++)
20	Solid adenocarcinoma	Negative	26 (+)	Negative
21	Solid adenocarcinoma	Negative	77.3 (+++)	Negative
22	Complex adenocarcinoma	Negative	29.2 (+)	Negative
23	Complex adenocarcinoma	Negative	94.7 (++)	Negative
24	Complex adenocarcinoma	Negative	29.2 (+)	32.3 (++)
25	Carcinosarcoma	Negative	16.1 (++)	Negative
26	Carcinosarcoma	15.96 (+++)	91.1 (+++)	Negative
27	Sebaceous adenocarcinoma	Negative	Negative	19.4 (+++)
28	Squamous cell carcinoma	Negative	33.2 (+)	43.5 (+++)
29	Hemangiopericytoma	Negative	99 (+++)	35.8 (+++)

**Table 2:** Distribution of ER*α* and PR in benign and malignant mammary tumors.

Tumor type	ER*α*+ PR+	ER*α*+ PR−	ER*α*− PR+	ER*α*− PR−	Total
Benign	**—**	**—**	6 (54.55%)	5 (45.45%)	11
Malignant	**—**	1 (5.56%)	9 (50%)	8 (44.44%)	18

Total	0	1	15	13	29

**Table 3:** Distribution of ER*β* and PR in benign and malignant mammary tumors.

Tumor type	ER*β*+ PR+	ER*β*+ PR−	ER*β*− PR+	ER*β*− PR−	Total
Benign	5 (45.45%)	4 (36.36%)	1 (9.09%)	1 (9.09%)	11
Malignant	6 (33.33%)	8 (44.44%)	3 (16.67%)	1 (5.56%)	18

Total	11	12	4	2	29

**Table 4:** Association of ER*β* with ER*α* and PR status in benign tumors (*n* = 11).

	ER*β* negative %	ER*β* positive %	*P* value
ER*α*			
Negative (%)	2 (18%)	9 (82%)	—
Positive (%)	0	0	

PR			
Negative (%)	1 (9%)	4 (36%)	>0.05
Positive (%)	1 (9%)	5 (46%)	

**Table 5:** Association of ER*β* with ER*α* and PR status in malignant tumors (*n* = 18).

	ER*β* negative %	ER*β* positive %	*P* value
ER*α*			
Negative (%)	4 (22%)	13 (72%)	>0.05
Positive (%)	0	1 (6%)	

PR			
Negative (%)	1 (6**%)**	8 (44**%**)	>0.05
Positive (%)	3 (17%)	6 (33%)	

## References

[1] Bostock DE (1986). Canine and feline mammary neoplasms. *British Veterinary Journal*.

[2] Hellmén E (2005). Complex mammary tumours in the female dog: a review. *Journal of Dairy Research*.

[3] Martín De Las Mulas J, Millán Y, Dios R (2005). A prospective analysis of immunohistochemically determined estrogen receptor *α* and progesterone receptor expression and host and tumor factors as predictors of disease-free period in mammary tumors of the dog. *Veterinary Pathology*.

[4] Nieto A, Peña L, Pérez-Alenza MD, Sánchez MA, Flores JM, Castaño M (2000). Immunohistologic detection of estrogen receptor alpha in canine mammary tumors: clinical and pathologic associations and prognostic significance. *Veterinary Pathology*.

[5] Millanta F, Calandrella M, Bari G, Niccolini M, Vannozzi I, Poli A (2005). Comparison of steroid receptor expression in normal, dysplastic, and neoplastic canine and feline mammary tissues. *Research in Veterinary Science*.

[6] Santen RJ, Yue W, Wang JP (2005). Potential mechanisms whereby estrogens induce breast cancer in women. *Breast Cancer Research*.

[7] Fuqua SAW (2001). The role of estrogen receptors in breast cancer metastasis. *Journal of Mammary Gland Biology and Neoplasia*.

[8] Sorenmo K (2003). Canine mammary gland tumors. *Veterinary Clinics of North America–Small Animal Practice*.

[9] Martín De Las Mulas J, Ordás J, Millán MY (2004). Immunohistochemical expression of estrogen receptor *β* in normal and tumoral canine mammary glands. *Veterinary Pathology*.

[10] Cork DMW, Lennard TWJ, Tyson-Capper AJ (2008). Alternative splicing and the progesterone receptor in breast cancer. *Breast Cancer Research*.

[11] Luna LG (1968). *Manual of Histologic Staining Methods of the Armed Forces Institute of Pathology*.

[12] Hampe JF, Misdorp W (1974). Tumours and dysplasias of the mammary gland. *Bulletin of the World Health Organization*.

[13] Fowler J, Cohen John L (1990). *Practical Statistics for Field Biology*.

[14] Jarvinen TAH, Pelto-Huikko M, Holli K, Isola J (2000). Estrogen receptor *β* is coexpressed with ER*α* and PR and associated with nodal status, grade, and proliferation rate in breast cancer. *American Journal of Pathology*.

[15] Moulton JE, Moulton JE (1990). Tumors of the mammary gland. *Tumors of Domestic Animals*.

[16] Thuróczy J, Reisvaag GJK, Perge E, Tibold A, Szilágyi J, Balogh L (2007). Immunohistochemical detection of progesterone and cellular proliferation in canine mammary tumours. *Journal of Comparative Pathology*.

[17] Diaz LK, Sneige N (2005). Estrogen receptor analysis for breast cancer: current issues and keys to increasing testing accuracy. *Advances in Anatomic Pathology*.

[18] Kuiper GGJM, Enmark E, Pelto-Huikko M, Nilsson S, Gustafsson JA (1996). Cloning of a novel estrogen receptor expressed in rat prostate and ovary. *Proceedings of the National Academy of Sciences of the United States of America*.

[19] Shao W, Brown M (2004). Advances in estrogen receptor biology: prospects for improvements in targeted breast cancer therapy. *Breast Cancer Research*.

[20] Hayashi SI, Eguchi H, Tanimoto K (2003). The expression and function of estrogen receptor *α* and *β* in human breast cancer and its clinical application. *Endocrine-Related Cancer*.

[21] Skliris GP, Munot K, Bell SM (2003). Reduced expression of oestrogen receptor *β* in invasive breast cancer and its re-expression using DNA methyl transferase inhibitors in a cell line model. *Journal of Pathology*.

[22] Illera JC, Pérez-Alenza MD, Nieto A (2006). Steroids and receptors in canine mammary cancer. *Steroids*.

[23] Geraldes M, Gärtner F, Schmitt F (2000). Immunohistochemical study of hormonal receptors and cell proliferation in normal canine mammary glands and spontaneous mammary tumours. *Veterinary Record*.

